# The Role of Global Femoral Offset in Total Hip Arthroplasty with High Hip Center Technique

**DOI:** 10.1111/os.13818

**Published:** 2023-07-31

**Authors:** Tianyu Yang, Boning Yang, Penghao Wang, Yu Qin, Guanchao You, Yunyi Shi, Ao Zhang, Dianlin Shen, Lei Guo

**Affiliations:** ^1^ Department of Orthopedics the First Hospital of China Medical University Shenyang China

**Keywords:** Developmental dysplasia of the hip, Global offset, High hip center, Total hip arthroplasty

## Abstract

**Objective:**

The high hip center (HHC) technique has been proposed for the treatment of patients with developmental dysplaisa of the hip (DDH) who have an acetabular bone defect. However, the importance of global femoral offset (FO) in the application of this technique has not been sufficiently appreciated. Our goals were to confirm that the HHC technique is feasible in the treatment of patients with DDH and to assess the function of global FO in this procedure.

**Methods:**

We retrospectively analyzed 73 patients who underwent total hip arthroplasty using high hip center technique for unilateral DDH at our hospital between January 2014 and June 2019. According to global FO, the patients were split into three groups: increased FO group (increment greater than 5 mm), restored FO group (restoration within 5 mm) and decreased FO group (reduction greater than 5 mm). Patients' medical records and plain radiographs were reviewed. One‐way ANOVA was used to compare radiographic outcomes and Harris hip score (HHS). Paired *t*‐test was used to assess preoperative and postoperative HHS and leg length discrepancy. Trochanteric pain syndrome, Trendelenburg sign and postoperative limp was evaluated with Fisher's exact test.

**Results:**

The average follow‐up time was 7.5 ± 1.4 years. The patients' HHS and leg length discrepancy were significantly improved (*p* < 0.05). In terms of vertical acetabular height, abductor arm, postoperative leg length difference, and acetabular cup inclination, there was no statistically significant difference between the three groups. At the last follow‐up, HHS was significantly higher in the restored FO group than in the decreased FO and increased FO groups. Trochanteric pain syndrome occurred in 15.0% and Trendelenburg sign and postoperative limp in 8.2% of all patients, respectively. Trochanteric pain syndrome, Trendelenburg sign and postoperative limp did not differ significantly across the three groups. One patient in increased FO group underwent revision for dislocation 6 years after surgery.

**Conclusion:**

The HHC technique is an alternative technique for total hip arthroplasty in patients with acetabular bone abnormalities, according to the results of the mid‐term follow‐up. Also, controlling the correction of the global femoral offset to within 5 mm may lead to better clinical outcomes.

## Graphical abstract

The high hip center technique seems to be an alternative technique for the treatment of patients with developmental dysplaisa of the hip who have an acetabular bone defect. It is crucial to restore the proper global femoral offset, corrections of up to 5 mm appear reasonable.

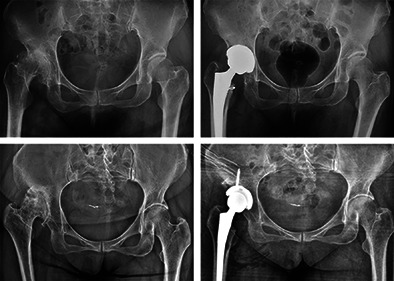

## Introduction

The high hip center (HHC) technique was introduced by Russotti and Harris in 1991,^1^ initially for total hip arthroplasty (THA) revision, and has since been increasingly used for complex acetabular deformities with partial or whole loss of the superior lateral border. Despite the possible biomechanical implications,^2^ the HHC technique has been brought forward as a viable substitute^3^ to address acetabular defects due to the complex and time‐consuming anatomical placement procedure of the socket cup, which usually requires structural bone grafting to supplement the bone deficiency^4^ and subtrochanteric shortening osteotomy to reduce the risk of nerve damage.^5^

The HHC technique has been adopted by many physicians in the treatment of patients with Crowe types II‐III developmental dysplaisa of the hip (DDH) and some revision cases with severe acetabular bone defects.^6^ Systematically reviewing 207 hips with HHC due to Crowe types II‐IV DDH, Stirling *et al*. found no differences between the HHC group and the control group in terms of revision incidence, intraoperative complications, or Harris hip score (HHS).^7^ Shen reported 85 hips using HHC technique without cup loosening at an average of 8.9 years after surgery.^6^ However, previous clinical studies mostly suffered from relatively small sample sizes, technical variations, different definitions of HHCs, and different demographics.^7^ Therefore, more rigorous study designs are needed to exclude the risk of bias as much as possible as well as more clinical studies to demonstrate the feasibility of HHC technique when treating patients with DDH.

After THA, a substantial correlation exists between femoral offset (FO) restoration and functional recovery. The effect of FO on the abductor and external rotator moment arms^8^ and soft‐tissue tension^9^ has been demonstrated. However, this measurement does not account for any potential acetabular cup positional effects. Hip function is positively impacted by medialization of hip center and a smaller body weight lever arm,^10^ while the height of hip center also affects abductor tension.^11^

Global FO was defined as the sum of the acetabular offset and FO.^12^ FO was defined as the distance from the center of the femoral head to the bisector of the long axis of the femur.^13^ Acetabular offset was defined as the distance from the center of the femoral head to the true floor of acetabulum.^14^ Worlicek *et al*. revealed the relationship between global FO and restoration of leg length and trochanteric pain syndrome.^15^ Mahmood *et al*. proved that global FO of more than 5 mm appeared to have a negative association with abductor muscle strength of operated hip.^16^ Overall, global FO has become an important perioperative parameter for THA. However, due to the funnel structure of the pelvis, an increase in acetabular offset will be inevitable when applying the HHC technique. The impact of an appropriate global FO on clinical prognosis has been confirmed by many scholars,^7^, ^17^ while few studies have examined the importance of restoring an appropriate global FO when applying HHC technique.

The objectives of this study were: (i) to evaluate the effect of cup medialization placement; (ii) to assess the effect of different global FO on clinical efficacy of patients; and (iii) to evaluate the revision rate after applying HHC technique in patients with DDH. To provide a reference for the treatment of patients with DDH in the presence of acetabular bone defects.

## Patients and Methods

### Criteria for Inclusion and Exclusion

The requirements for inclusion were: (i) individuals above the age of 18 with unilateral DDH; (ii) patients who had cementless THA at our hospital between January 2014 and June 2019 and were treated by a single surgeon; (iii) at the HHC (22 mm above the inter‐teardrop line), the acetabular cup was positioned; (iv) outcome measures included the global FO, cup position and inclination, abductor lever arm (ALA), leg length discrepancy (LLD), HHS, Trendelenburg sign, postoperative limp, trochanteric pain syndrome and survivorship; and (v) retrospective study.

The exclusion standards comprised: (i) patients who have undergone revision surgery; (ii) patients with a history of neuromuscular illness; and (iii) presence of disease in the contralateral hip.

### Patients

We conducted a retrospective study of a case series after receiving consent from the institutional review board (approval number: AF‐SOP‐07‐1.1‐01). From our departmental database, we identified 79 patients diagnosed with DDH with the acetabular cup positioned at the HHC, of which the threshold was established as 22 mm above the inter‐teardrop line. One patient died of an unrelated cause to the procedure at 6 years after surgery and five patients were lost to follow‐up. All the other patients completed their clinical exams and questionnaires. Consequently, 73 patients could ultimately participate in this trial.

The Crowe classification placed 51 patients in the type II category, 18 patients in the type III category, and four patients in the type IV category. Ten patients had a background of previous operations: two patients had open reductions, two had femoral derotational osteotomies, one had a hip arthroscopic surgery, and five had hip shelf procedures. These anatomical alterations resulting from previous surgical history were carefully evaluated preoperatively or intraoperatively and did not influence our intraoperative decision. (Table 1).

**TABLE 1:** Demographics of the patients

Demographic	Decreased FO group	Restored FO group	Increased FO group	Statistical value	
Number of patients	23	25	25		
Age (years)*	55.8 ± 9.1	57.9 ± 8.4	58.1 ± 7.1	*F* = 0.548	*p* = 0.581
Gender (*n*)				*X* ^2^ = 0.572	*p* = 0.751
Male	7 (30.4%)	8 (32%)	10 (40%)		
Female	16 (69.6%)	17 (68%)	15 (60%)		
Height (cm)*	162.5 ± 6.5	164.3 ± 9.7	165.1 ± 8.5	*F* = 0.608	*p* = 0.546
BMI (kg/m^2^)*	23.19 ± 3.43	23.79 ± 3.55	23.68 ± 2.78	*F* = 0.318	*p* = 0.728
Crowe classification				*X* ^2^ = 1.587	*p* = 0.823
Type II	14 (60.8%)	19 (76%)	18 (72%)		
Type III	7 (20.5%)	5 (20%)	6 (24%)		
Type IV	2 (8.7%)	1 (4%)	1 (4%)		
Surgical History	4 (17.3%)	3 (12.0%)	3 (12.0%)	*X* ^2^ = 0.387	*p* = 0.834

Abbreviations: BMI, body mass index; FO, femoral offset.

* The values were given as the mean and standard deviation.

### Groups According to the Global FO

These patients were divided into three groups based on their postoperative global FO measurements of the operated hip in comparison to the contralateral hip on plain radiographs: increased FO group (increment greater than 5 mm), restored FO group (restoration within 5 mm) and decreased FO group (reduction greater than 5 mm).

### Surgical and Placement of Prosthesis

All surgeries were carried out utilizing a Moore posterior approach while the patient was in the lateral position and under double‐block anesthesia. Intentional medial placement and cup orientation adjustment were performed with the goal of achieving at least 70% bone cup surface contact. When satisfactory stability was attained, partial covering of the superior lateral margin was deemed acceptable. Following surgery, rivaroxaban antithrombotic prophylaxis was started for all patients. On the first postoperative day, patients were allowed to fully bear weight and began the same postoperative rehabilitation regimen. Table 2 provided thorough information on the acetabular and femoral components and forms of bearing.

**TABLE 2:** Types of bearing used in all patients and designs of acetabular and femoral components

Indexes	Decreased FO group	Restored FO group	Increased FO group
Acetabular component			
Pinnacle (DePuy, Warsaw, IN, USA)	20 (86.9%)	22 (88.0%)	23 (92.0%)
Betacup (Link, Hamburg, Germany)	3 (13.1%)	3 (12.0%)	2 (8.0%)
Femoral stem			
Corail (DePuy, Warsaw, IN, USA)	7 (30.4%)	10 (40.0%)	10 (40.0%)
Trilock (DePuy, Warsaw, IN, USA)	12 (52.1%)	12 (48.0%)	11 (44.0%)
S‐ROM (DePuy, Warsaw, IN, USA)	1 (4.4%)	‐	2 (8.0%)
Ribbed (Link, Hamburg, Germany)	3 (13.1%)	3 (12.0%)	2 (8.0%)
Bearing type			
Ceramic on ceramic	16 (69.6%)	18 (72.0%)	16 (64.0%)
Ceramic on polyethylene	5 (21.7%)	6 (24.0%)	9 (36.0%)
Metal on polyethylene	2 (8.7%)	1 (4.0%)	‐

Abbreviation: FO, femoral offset.

### Radiographic Evaluation

**FIGURE 1:**
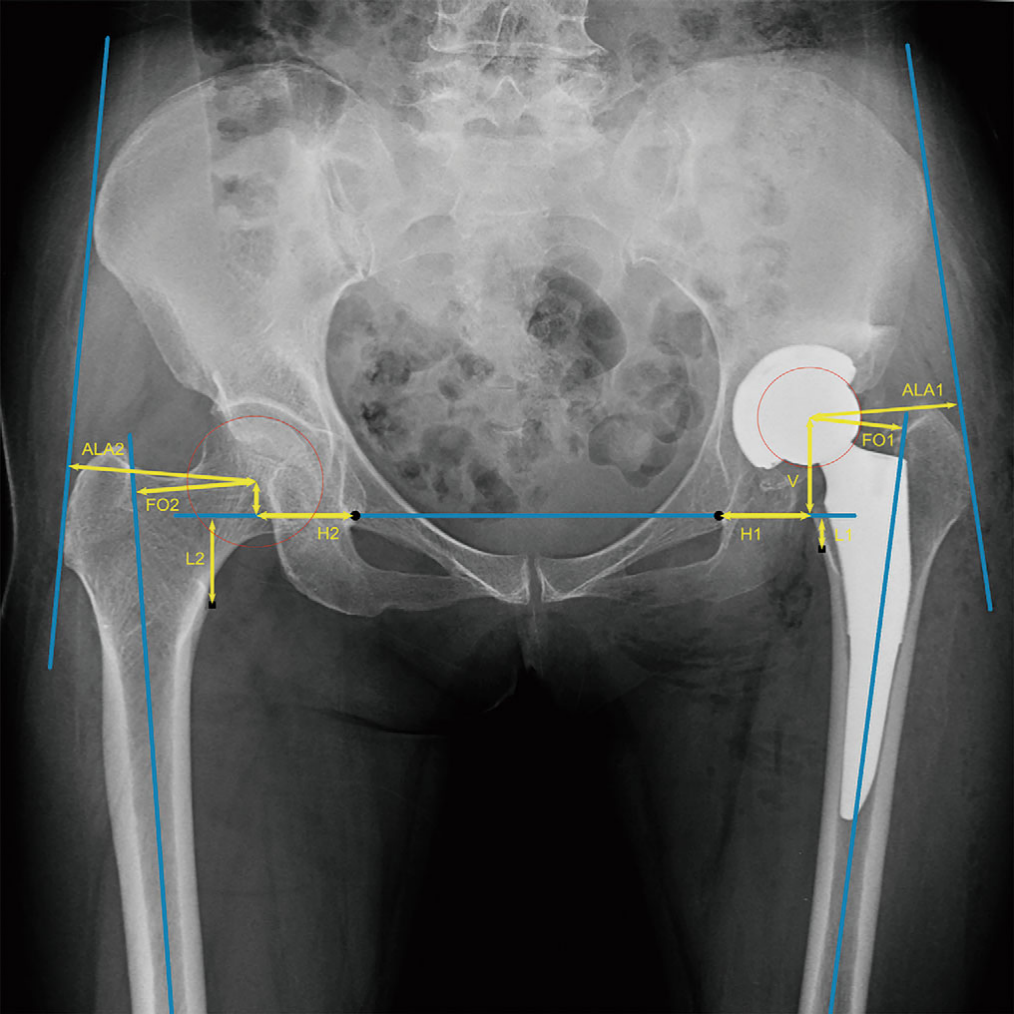
Diagram radiography measurement. Black dots represented teardrops and black squares represented small rotor vertices. ALA, abductor lever arm; FO, femoral offset; H: horizontal distance; V, vertical distance; L, leg length; global FO = H + FO; **△**horizontal distance of hip center = H1 – H2; LLD = L1 – L2.

Anteroposterior pelvic radiographs were used to evaluate each patient's radiological condition on the day of surgery and 3 months beforehand. Calibration using actual femoral head prosthesis dimensions was used to assess magnification. To compare horizontal distance of hip center, global FO and leg length of the operated side to those of the unoperated side, the measurement was repeated bilaterally. Positive values were obtained when the surgical hip side's value was higher than the non‐operative hip side, while negative values represented the reverse. The precision scale was 0.1 mm. After obtaining consistency of measurement criteria, radiological measurements were performed by two investigators independently and results were averaged over the data obtained from two measurements. (Figure 1).

#### Global FO and Medialization

The global FO was calculated by adding the distance between the longitudinal axis of the femur and the center of the femoral head to the horizontal distance of hip center in relation to the teardrop. Medialization was defined as the difference in the horizontal distance of hip center between bilateral hip.

#### Cup Inclination and Position

The horizontal and vertical distances between the hip center and acetabular teardrop were used to define the cup's position. The difference between the vertical distance from hip center on the operated side and the opposite side is defined as extent of the upward movement. The connecting line to the cup's rim's borders and the interteardrop line's abduction angle were used to establish the cup's inclination.

#### Leg Length Discrepancy and Abductor Lever Arm

The was calculated as the difference in length between the lesser trochanter's tip and the line bridging the two sides' caudal edges of the teardrop. The greater trochanter's lateral portion and the anterosuperior iliac crest were joined by a line, and this was used to measure the ALA.

### Clinical Assessment

The HHS, trochanteric pain syndrome, Trendelenburg sign, and postoperative limp were used in our clinical evaluation of each patient.

#### Note on HHS

The HHS was developed as a method to assess the outcomes of mold arthroplasty. Pain, function, deformity, and range of motion are the four dimensions covered by the HHS. The HHS has a total of 10 items, with a maximum score of 100 points. For purposes of interpretation, score ranges between 100 and 90 reflect great results, 90 and 80 points, decent results, 80 and 70 points, and sub‐70 points, poor results, respectively.

#### Criteria for Trendelenburg Sign and Postoperative Limp

The Trendelenburg sign was used to assess the strength of the gluteus medius muscle. The capacity of the patient to elevate their non‐weight‐bearing side pelvis to a high position and maintain it for at least 5 s when the examiner instructs them to lift one leg off the ground while flexing their hip was described as a negative Trendelenburg sign. Any discernible lateral imbalance in the pelvic movement during walking was classified as a limp.

#### Trochanteric Pain Syndrome

Greater trochanter discomfort on palpation and painful hip active abduction were the two characteristics of trochanteric pain syndrome. An impartial observer who was blind to the radiological findings conducted a clinical evaluation to determine whether trochanteric pain syndrome was present.

### Statistical Analysis

The mean and standard deviation were used to express continuous data including demographics, radiographic measures, and HHS. One‐way ANOVA was used to analyze the differences of quantitative data between the three groups. Paired *t*‐test was used to assess the comparison of preoperative and postoperative HHS and LLD in all patients. The comparison of trochanteric pain syndrome, surgical history, Trendelenburg sign and postoperative limp was evaluated with Fisher's exact test. Revision for any cause was established as the criterion for survival. To calculate the probability of survival in the three groups, Kaplan–Meier analysis was used. The log‐rank test was used to compare the equality of the survival distributions between the two groups. The cutoff for significance was *p* < 0.05. Software SPSS Version 25.0 software (IBM, Armonk, NY, USA) was used for all analyses.

## Results

### Results of Follow‐up and Overall

All 73 patients had a mean follow‐up time of 7.5 ± 1.4 years (with a range of 5.0 to 11.0); the decreased FO group, restored FO group and increased FO groups had follow‐up times of 7.4 ± 1.4 years, 7.7 ± 1.3 years and 7.5 ± 1.5 years, respectively (*p* = 0.785). The average operation time in the decreased FO group was 2.0 ± 0.3 h, and the average intraoperative blood loss was 573 ± 105 mL. The average surgery time in the restored FO group was 1.9 ± 0.2 h, and the average intraoperative bleeding was 588 ± 99 mL. The average surgery time in the increased FO group was 2.0 ± 0.3 h, and the average intraoperative bleeding was 548 ± 72 mL. Between three groups, there was no discernible difference in the length of operation (*p* = 0.193) or intraoperative bleeding (*p* = 0.313).

### Radiographic Results

#### Global FO, LLD and ALA

In the decreased FO group, the global FO, LLD and ALA were −9.8 ± 3.2 mm, 0.2 ± 5.8 mm and 47.9 ± 5.7 mm. In the restored FO group, those were 0.8 ± 2.8 mm, 1.6 ± 5.3 mm and 48.5 ± 5.6 mm. In the increased FO group, those were 9.2 ± 2.5 mm, 3.2 ± 3.8 mm and 48.5 ± 5.8 mm. Therefore, except for the global FO, LLD and ALA did not significantly differ across the groups (*p* = 0.130 and *p* = 0.920, respectively). (Table 3).

**TABLE 3:** Postoperative radiographic evaluation

Evaluation parameter	Decreased FO group*	Restored FO group*	Increased FO group*	Statistical value	
Global femoral offset (mm)	−9.8 ± 3.2	0.8 ± 2.8	9.2 ± 2.5		
Vertical distance (mm)	26.7 ± 3.8	25.9 ± 2.8	25.4 ± 2.4	*F* = 0.920	*p* = 0.403
Horizontal distance (mm)	30.4 ± 3.1	31.7 ± 3.8	33.6 ± 3.9	*F* = 4.662	*p* = 0.012
Abductor lever arm (mm)	47.9 ± 5.7	48.5 ± 5.6	49.8 ± 5.3	*F* = 0.676	*p* = 0.511
Preoperative leg length discrepancy (mm)	−20.7 ± 5.1	−21.7 ± 5.9	−18.9 ± 3.8	*F* = 2.048	*p* = 0.136
Postoperative leg length discrepancy (mm)	0.2 ± 5.8	1.6 ± 5.3	3.2 ± 3.8	*F* = 2.113	*p* = 0.130
Cup inclination (degree)	37.8 ± 4.7	36.2 ± 4.5	37.0 ± 4.3	*F* = 0.738	*p* = 0.481

Abbreviation: FO, femoral offset.

* The values were given as the mean and standard deviation.

#### Cup Position and Medialization

**FIGURE 2:**
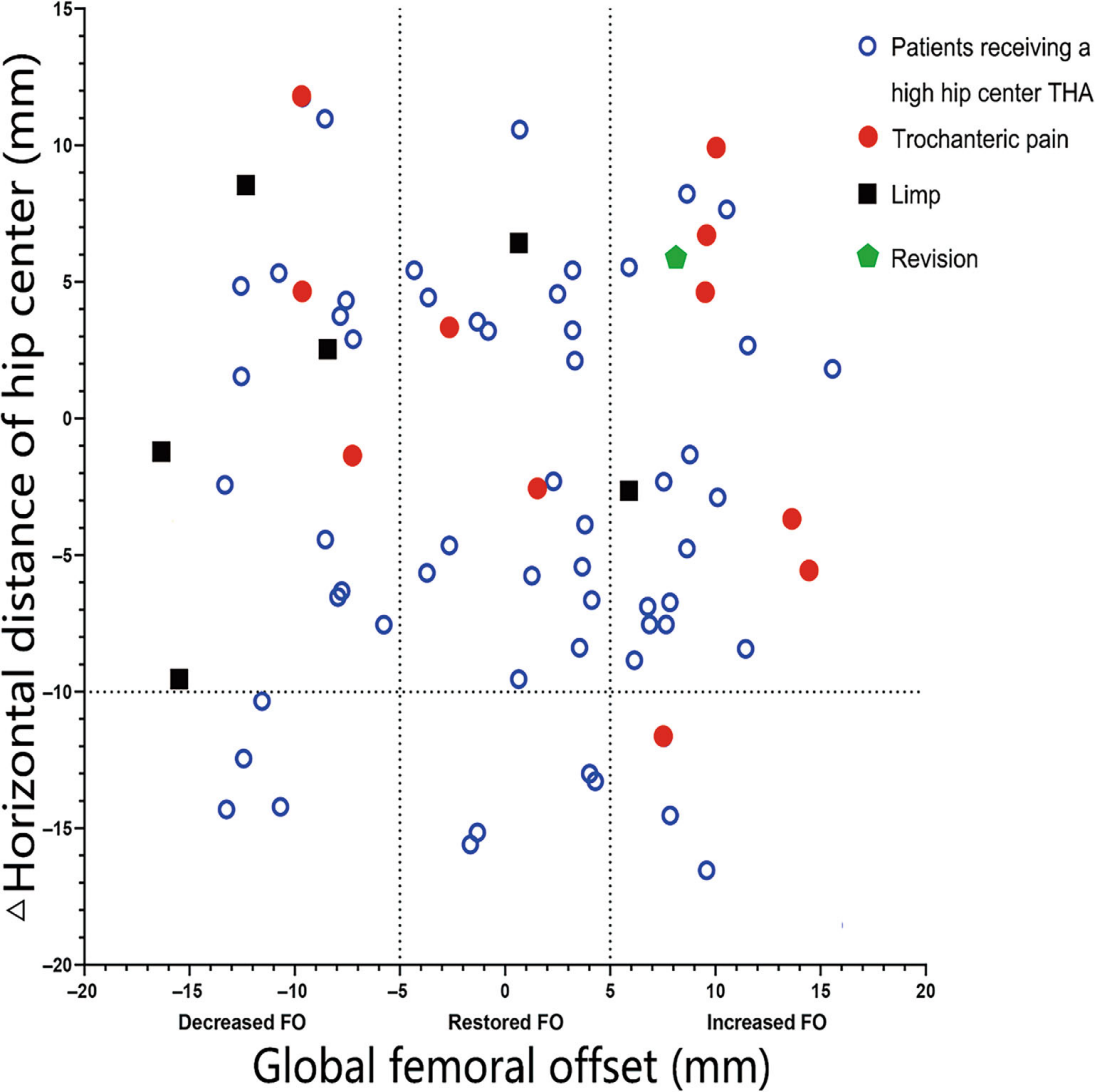
Scatter‐gram of cup position in the decreased femoral offset (FO), restored FO and increased FO group. △Horizontal distance of hip center was defined as difference between the surgical side and the contralateral side. THA, total hip arthroplasty.

Scatter diagram showing the distribution of the hip joint center relative to the anatomical center (Figure 2). The mean position of the cup from the inter‐teardrop was 26.7 ± 3.8 mm vertically (upward movement was 14.4 ± 2.1 mm) and 30.4 ± 3.1 mm horizontally in the decreased FO group, 25.9 ± 2.8 mm vertically (upward movement was 15.2 ± 1.9 mm) and 31.7 ± 3.8 mm horizontally in the restored FO group and 25.4 ± 2.4 mm vertically (upward movement was 14.5 ± 2.0 mm) and 33.6 ± 3.9 mm horizontally in the increased FO group. Therefore, the horizontal position between the groups was significantly different (*p* = 0.012), but the vertical position (*p* = 0.403) and upward movement (*p* = 0.351) between the groups did not differ significantly. The mean cup inclination of the decreased FO, restored FO and increased FO groups were 37.8 ± 4.7°, 36.2 ± 4.5° and 37.0 ± 4.3°, respectively (*p* = 0.479). In the decreased FO group, four hips showed a lateralization over 10 mm, as did four hips in the restored FO group and three hips in the increased FO group (*p* = 0.920).

### Clinical Results

#### Overall Postoperative Outcomes

**FIGURE 3:**
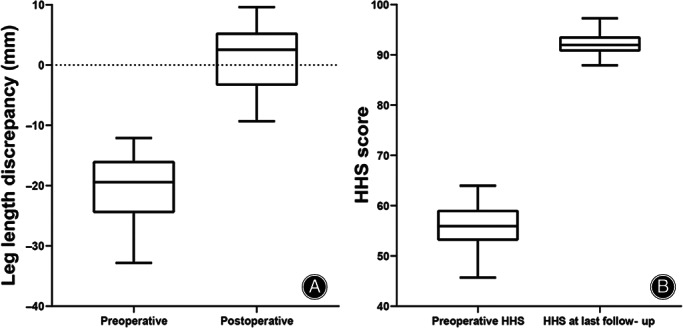
(A) The mean leg length discrepancy for all patients. (B) The mean Harris Hip Score (HHS) for all patients.

The mean LLD for all 73 patients was improved from −20.4 ± 5.1 mm to 1.4 ± 5.3 mm and the mean HHS improved from 55.9 ± 4.0 points to 92.2 ± 2.1 points. The HHS (*p* < 0.05) and LLD (*p* < 0.05) at the time of final follow‐up were significantly improved. (Figure 3).

#### Harris Hip Score

**FIGURE 4:**
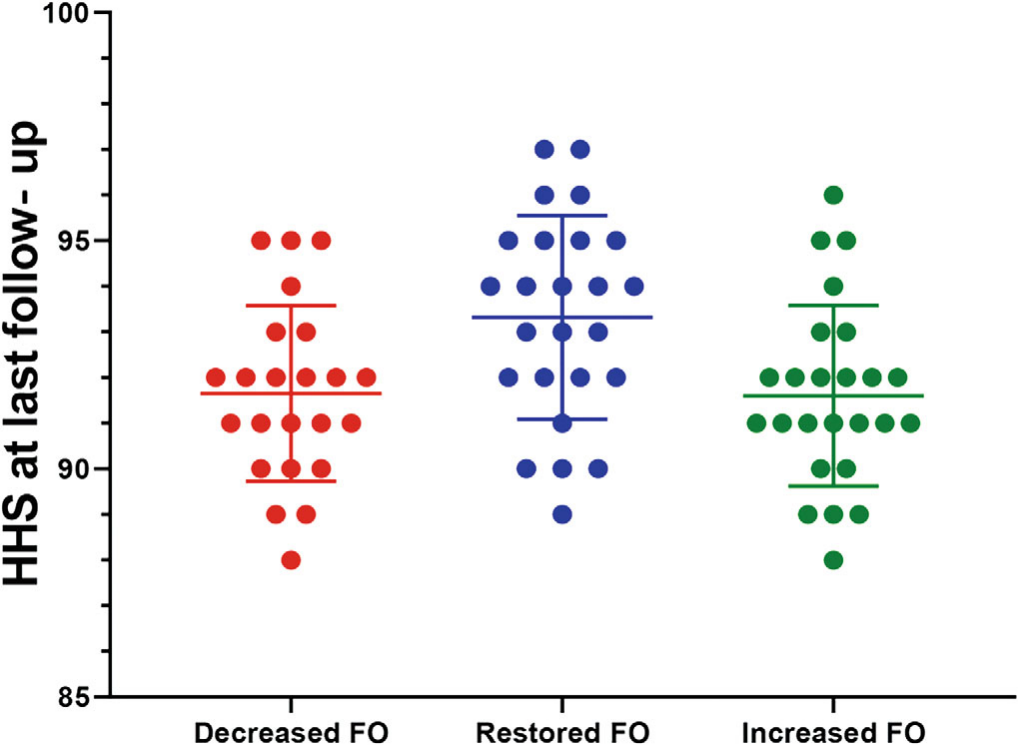
The postoperative Harris hip score (HHS) in the final follow‐up of decreased femoral offset (FO), restored FO and increased FO group.

The preoperative HHS of the decreased FO, restored FO and increased FO groups were 56.2 ± 4.1, 55.0 ± 4.4 and 56.7 ± 3.6, respectively (*p* = 0.317). The postoperative HHS in the final follow‐up of restored FO was 93.3 ± 2.2, which was significantly higher than that of the decreased FO group (91.6 ± 1.9) (*p* = 0.011) and the increased FO group (91.5 ± 2.0) (*p* = 0.007). There was no significant difference between the decreased FO and increased FO groups (*p* = 0.900). (Figure 4).

#### Trendelenburg Sign and Postoperative Limp

Of the 73 patients, four patients in decreased FO group, one patient in restored FO group and one patient in increased FO group showed a positive Trendelenburg sign and presented with a limp. More patients in the decreased FO group were positive for Trendelenburg's sign than in the restored FO and decreased FO groups, but there was no significant difference between them (*p* = 0.267). (Table 4).

**TABLE 4:** Clinical evaluation

Parameters	Decreased FO group	Restored FO group	Increased FO group	Statistical value	
Preoperative HHS*	56.2 ± 4.1	55.0 ± 4.4	56.7 ± 3.6	*F* = 1.167	*p* = 0.317
HHS at last follow‐up*	91.6 ± 1.9	93.3 ± 2.2	91.5 ± 2.0	*F* = 5.614	*p* = 0.005
Positive Trendelenburg sign	4 (17.3%)	1 (4.0%)	1 (4.0%)	*X* ^2^ = 3.063	*p* = 0.267
Postoperative limp	4 (17.3%)	1 (4.0%)	1 (4.0%)	*X* ^2^ = 3.063	*p* = 0.267
Trochanteric pain syndrome	3 (13.0%)	2 (8.0%)	6 (24.0%)	*X* ^2^ = 2.419	*p* = 0.299

Abbreviation: FO, femoral offset; HHS, Harris Hip Score.

* The values were given as the mean and standard deviation.

#### Trochanteric Pain Syndrome

The prevalence of trochanteric pain syndrome in our cohort was 15.0% (11/73). Six patients in the increased FO group presented with trochanteric pain syndrome, more than in the restored FO (two patients) and the decreased FO group (three patients). No significant difference was shown regarding trochanteric pain syndrome between the three groups (*p* = 0.299). (Table 4).

#### Patient's Revisions and Kaplan–Meier Survival Rate

**FIGURE 5:**
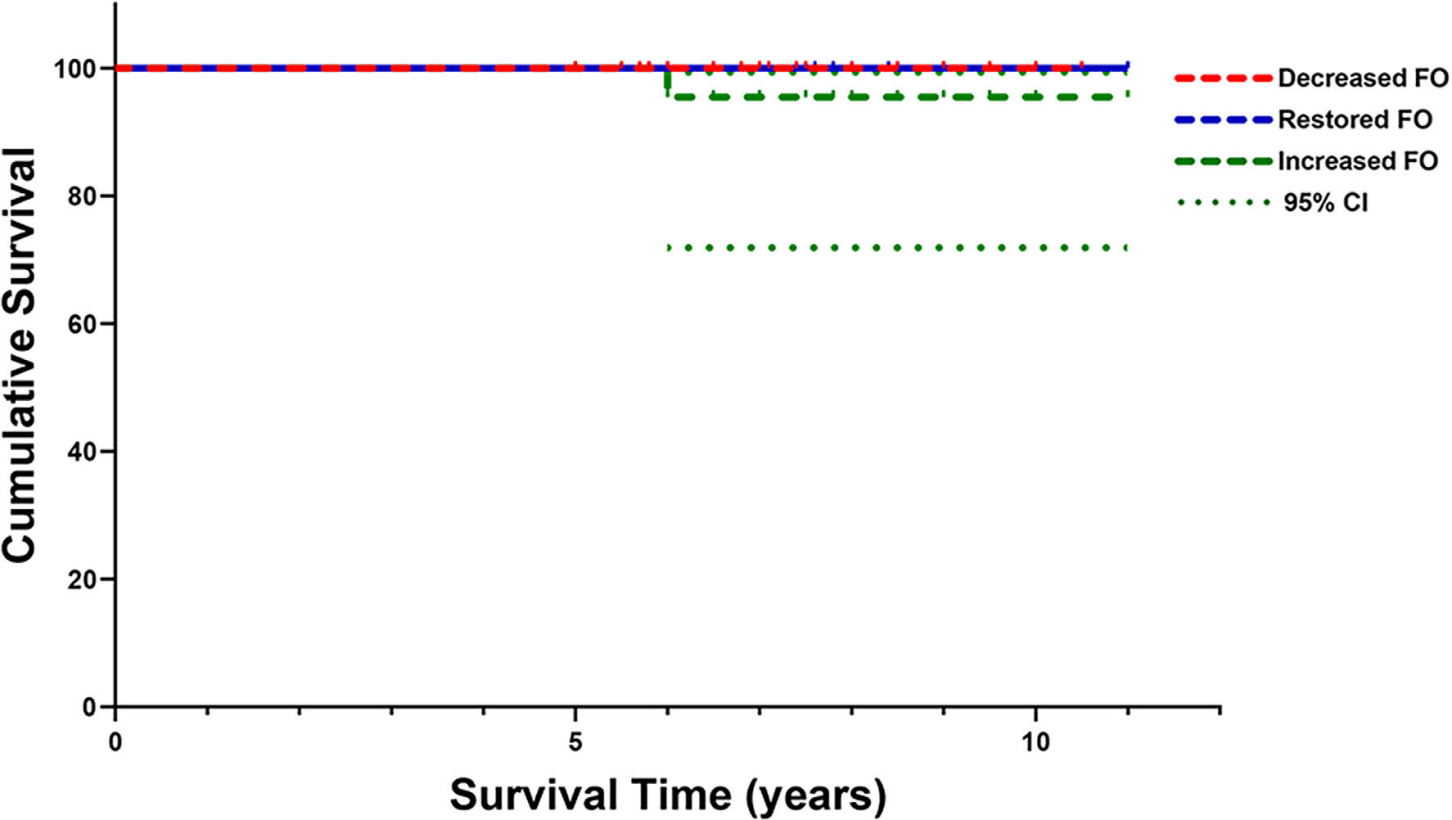
For decreased femoral offset (FO), restored FO and increased FO group, the Kaplan–Meier survival curve with revision for any cause was displayed. CI, confidence interval.

**FIGURE 6:**
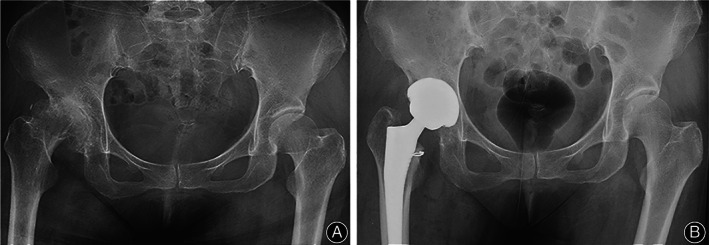
Radiographic image of a 52‐year‐old female patient. (A) Preoperative diagnosis of Crowe II hip dysplasia. (B) Measurements showed a right hip center with a height of 26.4 mm and no acetabular cup loosening or osteolysis on radiographic evaluation after 8.5 years.

Among the 73 hips, the overall revision rate was 1.3% (1/73). Six years following surgery, one patient in the increased FO group was revised due to a dislocation. The Kaplan–Meier survival rates at last follow‐up were similar in the three groups (*p* = 0.347) with revision for any cause as the end point, 100% in the decreased FO group, 100% in the restored FO group and 96% (95% CI, 72%–99%) in the increased FO group. (Figures 5 and 6).

## Discussion

In the current study, all patients who applied the HHC technique showed significant improvement in LLD and HHS. We found that the HHS was highest in the restored FO group compared to increased FO group and decreased FO group, while there were no significant differences in postoperative complications such as trochanteric pain syndrome, revision rate, Trendelenburg sign and postoperative limp. This study suggested that restoring the global FO within 5 mm should be considered for better clinical outcomes when applying the HHC technique to treat patients with DDH.

### Medial Placement of the Cup

In our study, the acetabular prosthesis was placed medially near the medial wall during the surgery. Watts *et al*. found in a retrospective study that acetabular reconstruction at <1 cm superior and 1 cm lateral to acetabular anatomy center had a lower rate of aseptic loosening and cup revision.^18^ None of the patients in the present study experienced issues such as liner wear, severe lateralization, or loosening. One possible explanation is that the medialized placement of the cup reduces the reaction force of the joint and decreases the muscle strength of the abductor muscle, and another explanation is that there is more bone volume around the cup placed in the HHC and the rate of bone fusion of the cup is higher.^6^ Most of the abductors in patients with DDH are atrophied, so restoring an appropriate global FO is especially important, and the medialized placement of the cup can offset some of the increased acetabular offset due to placement of the cup at a HHC, which allows the clinician to better adjust the patient's global FO. Due to the funnel shape of the pelvis, an elevated center of hip will increase the difficulty of medializing the placement of the cup, as well as the potential for low medial wall bone volume, and therefore needs to be evaluated carefully when applied.^19^

### Clinical Efficacy

According to biomechanical research, abductor strength is negatively connected with the height of hip center, and increasing height causes a decrease in abductor length.^20^ As a result, it is crucial to restore proper global FO and abductor strength arms when using the high center of rotation technique. Proper femoral stem and appropriate head/neck length are also required to correct lower extremity leg length discrepancies in order to prevent claudication, which calls for careful planning and balancing on the part of the orthopedic surgeon.^21^ In our study, only 8.2% of patients developed Trendelenburg sign and postoperative limp. This was similar to the results (4.4%) of the large sample study by Iorio *et al*.^22^

The greater trochanter is the most noticeable part of the proximal lateral hip, and pain in the lateral trochanter may result from iliotibial band stress over the gluteus medius and minimus tendons and related bursae.^23^ After a total hip replacement, a typical consequence is ongoing pain in the area of the greater trochanter. Most of this pain can be treated by non‐surgical means.^24^ Numerous studies have shown that the surgical approach may have an impact on the incidence of this condition,^22^, ^25^ while Worlicek *et al*. found that patients with absolute deviations in combined biomechanical recovery of leg length, femoral and acetabular offset of more than 5 mm frequently experienced symptoms of trochanteric pain syndrome.^15^ In the present study, although the increased FO group had a higher incidence of pain in the current investigation (24.0%), there was no statistically significant difference between the three groups (*p* = 0.299), suggesting that a greater sample size could be necessary to obtain meaningful results. It is important to emphasize that the restored FO group scored much higher than the decreased FO and increased FO groups according to the HHS. This was comparable to the outcomes of Mahmood *et al*.^16^ It demonstrates how crucial it is to reinstate the correct FO when utilizing the HHC technique.

### Survival Rates of Implants

HHC was linked to a higher frequency of dislocation and a lower incidence of neurological sequelae, according to a systematic review with a sizable sample size.^7^ In our study, only the increased FO group had just one reconstructed hip because of a dislocation. In the increased FO group, the implants survival rate was 96% (95% CI, 72%–99%). The reason for our lower overall revision rate may be due to fact that the main body of patients in our study were Crowe types II‐III DDH patients and did not include patients who had revision surgery. Also, we positioned the center of HHC at 22 mm from the interteardrop line, which is lower than the definition of Nawabi *et al*. (28 mm).^26^ This may be one of the reasons why the surgeries did not fail due to acetabular loosening. Although from a mechanical point of view, the lower the height of the center of hip, the better the biomechanical balance of the hip joint and thus the lower the rate of loosening.^27^ However, in the study by Murayama *et al*., the average vertical distance of the hip center was 26.8 mm and the survival rate of the acetabular prosthesis was almost 100%,^28^ so the relationship between acetabular placement height and revision rate needs to be further investigated. Overall, the use of HHC technique in THA represents an alternative approach for treating DDH patients with acetabular bone defects.

### Strengths and Limitations

In the present study, we considered the importance of Global FO while applying the HHC technique, which has received little attention in previous studies. We also conducted a retrospective study of some of important clinical complications and collected data of research value. Also, this research has several limitations. First, our sample size was relatively small, and a longer follow‐up period is necessary for the HHC technique to be validated. Second, as opposed to CT, our measures were based on plain radiographs, which are less accurate. Third, we only roughly calculated the strength of the abductor muscles using the Trendelenburg sign rather than quantifying it. Fourth, we used HHS to measure patients' postoperative quality of life; possibly we should have used a more thorough evaluation system. Fifth, some preoperative strategies may be required to screen out patients who may have potential trochanteric pain syndrome.

### Conclusion

When acetabular bone defects are present in patients with DDH, we believed that HHC technique was an additional and beneficial procedure for THA. Additionally, it appeared that global FO recovery was crucial for patients' quality of life following surgery; however, larger comparative studies are required to fully understand how changes in the FO affect some complications.

## Author Contributions

Tianyu Yang: designing the study, analyzing the data, writing the manuscript; Lei Guo: designing the study, editing the manuscript; Penghao Wang, Boning Yang, Yunyi Shi, Yu Qin and Guanchao You: collecting the data, analyzing the data, reviewing the manuscript; Dianlin Shen and Ao Zhang: reviewing the literature. All authors have read and approved the final version of this manuscript.

## Funding

This work was supported by the National Natural Science Foundation of China (No. 81971322).

## Conflict of Interest

The authors declare that they have no conflict of interest.

## Ethics Approval and Consent to Participate

The study protocol was accepted by the ethics committee of our hospital, the First Hospital of China Medical University. This was a retrospective study thus the informed consent requirement was waived.
